# Syk Regulates Neutrophilic Airway Hyper-Responsiveness in a Chronic Mouse Model of Allergic Airways Inflammation

**DOI:** 10.1371/journal.pone.0163614

**Published:** 2017-01-20

**Authors:** Sepehr Salehi, Xiaomin Wang, Stephen Juvet, Jeremy A. Scott, Chung-Wai Chow

**Affiliations:** 1 Division of Respirology, Department of Medicine, Faculty of Medicine, University of Toronto, Toronto, Ontario, Canada; 2 Division of Medical Sciences, Northern Ontario School of Medicine, Thunder Bay, Ontario, Canada; 3 Southern Ontario Center for Atmospheric Aerosol Research, Faculty of Applied Sciences, University of Toronto, Toronto, Ontario, Canada; 4 Division of Occupational and Environmental Health, Dalla Lana School of Public Health, University of Toronto, Toronto, Ontario, Canada; 5 Department of Health Sciences, Faculty of Health and Behavioural Sciences, Lakehead University, Thunder Bay, Ontario, Canada; 6 Multi-Organ Transplant Programme, University Health Network, Toronto, Ontario, Canada; Forschungszentrum Borstel Leibniz-Zentrum fur Medizin und Biowissenschaften, GERMANY

## Abstract

**Background:**

Asthma is a chronic inflammatory disease characterized by airways hyper-responsiveness (AHR), reversible airway obstruction, and airway inflammation and remodeling. We previously showed that Syk modulates methacholine-induced airways contractility in naïve mice and in mice with allergic airways inflammation. We hypothesize that Syk plays a role in the pathogenesis of AHR; this was evaluated in a chronic 8-week mouse model of house dust mite (HDM)-induced allergic airways inflammation.

**Methods:**

We used the Syk^flox/flox^//rosa26CreER^T2^ conditional Syk knock-out mice to assess the role of Syk prior to HDM exposure, and treated HDM-sensitized mice with the Syk inhibitor, GSK143, to evaluate its role in established allergic airways inflammation. Respiratory mechanics and methacholine (MCh)-responsiveness were assessed using the flexiVent^®^ system. Lungs underwent bronchoalveolar lavage to isolate inflammatory cells or were frozen for determination of gene expression in tissues.

**Results:**

MCh-induced AHR was observed following HDM sensitization in the Syk-intact (Syk^flox/flox^) and vehicle-treated BALB/c mice. MCh responsiveness was reduced to control levels in HDM-sensitized Syk^del/del^ mice and in BALB/c and Syk^flox/flox^ mice treated with GSK143. Both Syk^del/del^ and GSK143-treated mice mounted appropriate immune responses to HDM, with HDM-specific IgE levels that were comparable to Syk^flox/flox^ and vehicle-treated BALB/c mice. HDM-induced increases in bronchoalveolar lavage cell counts were attenuated in both Syk^del/del^ and GSK143-treated mice, due primarily to decreased neutrophil recruitment. Gene expression analysis of lung tissues revealed that HDM-induced expression of IL-17 and CXCL-1 was significantly attenuated in both Syk^del/del^ and GSK143-treated mice.

**Conclusion:**

Syk inhibitors may play a role in the management of neutrophilic asthma.

## Introduction

Asthma is characterized by chronic airway inflammation, hyperresponsiveness (AHR), remodelling, and reversible airflow obstruction [1,2]. Despite the availability of effective anti-inflammatory and bronchodilator therapies, the incidence of asthma has grown worldwide [3–7]. In developed countries, asthma remains an important health issue that is associated with a high economic burden and disease morbidity.

Multiple genetic and environmental factors, i.e., exposure to allergens, respiratory viruses and pollutants, are associated with development of asthma and episodic exacerbations [1,2]. It is increasingly recognized that asthma manifests as multiple phenotypes that can be classified by different factors such as disease severity, predominant inflammatory phenotype (i.e., atopic, non-atopic, eosinophilic and/or neutrophilic), age of onset and response to corticosteroid therapy [8–15]. These phenotypes are regulated by distinct cellular and molecular pathways, thus providing impetus to develop better targeted therapies for distinct subpopulations.

Syk, a tyrosine kinase that plays key roles in innate immunity and inflammation, has recently been implicated in the pathogenesis of asthma in animal models of allergic airways inflammation [16–21]. Previous studies, in acute and subacute models of allergic airways inflammation [17,19,20], identified a role for Syk in modulating mast and dendritic cell function. Recently, we reported a role for Syk in a chronic murine model of ovalbumin (OVA)-induced allergic airways disease; we observed preferential increase in Syk expression in airway epithelia, and that a single dose of intratracheally delivered Syk-specific inhibitor effectively abrogated the methacholine (MCh)-induced AHR, without affecting leukocyte recruitment to the lungs [21]. A second study in naïve mice showed the MCh-induced airway contraction to be Syk-dependent, both *in vivo* and *ex vivo* [22]. Together, these observations suggest that Syk could play multiple roles in the pathogenesis of asthma; by modulating airway inflammation and immunity, with an additional role in mediating airway contractility.

We hypothesized that Syk plays a role in the pathogenesis of asthma by modulating AHR and airway inflammation, and evaluated this hypothesis using a chronic mouse model of allergic airways inflammation.

## Materials and Methods

### Induction of Syk knockout in the Syk^flox/flox^//rosa26CreER^T2^ mice, development of the chronic HDM model of allergic airways inflammation and treatment with the Syk inhibitor, GSK143

All animal protocols were approved by the University of Toronto Faculty Advisory Committee on Animal Services and Toronto General Research Institute Advisory Committee on Animal Services, and conducted in accordance with the guidelines of the Canadian Council on Animal Care (CCAC). The animals were housed in a CCAC-approved research facility. The health status of animals was monitored daily by the Research Facility staff and by the individual investigators conducting the studies. No animals became ill or died prior to the study endpoint. Mice were euthanized using anesthetic overdose with ketamine/xylazine followed by cervical disclocation according to institutional policy.

The inducible Syk^flox/flox^//rosa26CreER^T2^ mouse strain (gift from Boehringer Ingelheim [23]), the tamoxifen induction protocol and characterization of the Syk knock-out phenotype have been described [22]. Control littermates were treated with 10% *v/v* ethanol in sunflower oil, the diluent for tamoxifen. Syk deletion persists for 12 weeks [22], a period that spans the duration of the current HDM sensitization and challenge protocol. All mice were genotyped prior to sensitization to confirm Syk deletion.

**Fig 1:**
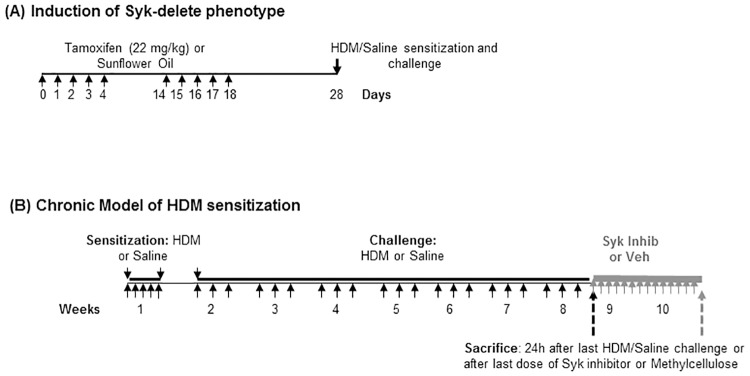
Time-lines of tamoxifen-induced knock-out of Syk, chronic HDM exposure models of allergic airways inflammation and treatment with Syk inhibitor. **(A)** *Induction of the Syk knock-out in the Syk*^*flox/flox*^*//rosa26CreER*^*T2*^ *mice*. Tamoxifen (Tam; 22 mg/kg) was administered by oral gavage. Control mice were gavaged with sunflower oil (SFo) as a vehicle control. **(B)** *Timelines of the chronic HDM-exposure models of allergic airways inflammation and treatment with Syk inhibitor*. In the Syk^flox/flox^//rosa26CreER^T2^ mice, HDM sensitization and challenge was initiated 10 days after the last treatment with tamoxifen or sunflower oil. Assessment of physiological endpoints occurred 24 h after the last dose of tamoxifen or sunflower oil. BALB/c and Syk^flox/flox^ mice were exposed to HDM for 8 weeks, and then treated daily for 2 weeks with GSK143 (30 mg/kg) by oral gavage or 1% methylcellulose (Veh) control, prior to assessment of the physiological responses 24 h later.

The chronic HDM-induced model of allergic airways inflammation was established by intranasal instillation of *Dermatophagoides pteronyssinus* crude extract (25 μg in 35μl normal saline) for 5 consecutive days, followed by intranasal challenge every other day for 8 weeks [24] (Fig 1). Control groups were challenged with 35μl of intranasal normal saline. Female BALB/c mice were randomized for oral gavage daily with GSK143 (30 mg/kg) or 1% methylcellulose (MC) as a vehicle control for two weeks after the 8-week HDM sensitization. The selectivity, potency and bioavailability of GSK2230413 (GSK143; a gift from GlaxoSmithKline) have been published [25].

Twenty-four hours after the last HDM or saline challenge (Syk^flox/flox^//rosa26CreER^T2^ mice) or dose of GSK143 or the methycellulose vehicle (BALB/c and Syk^flox/flox^ mice), mice were anesthetized with ketamine/xylazine (10 mg/kg and 50 mg/kg, respectively) for pulmonary function testing using the flexiVent^®^ (Scireq Inc., Montréal, PQ) [21,26–28]. Rocuronium (1.8–4.8 mg/kg) was given during pulmonary function testing to prevent respiratory drive artifact. Following assessment of pulmonary function, a subset of mice underwent bronchoalveolar lavage (BAL); BAL was conducted in heart-beating live, anesthetized mice. Mice were then euthanized with an overdose of ketamine/xylazine, whole blood samples were collected by cardiac puncture, and the lungs were then harvested, without further perfusion. BAL total and differential cell counts were performed as previously described [21]. Additional subsets of mice that had not undergone BAL were used for histological analysis, or isolation of pulmonary leukocytes for fluorescence activated cell sorting (FACS) analysis.

### Histology of mouse lung sections

Lung tissue sections were inflation-fixed to 25 cm H_2_O in 4% paraformaldehyde [28]. Hematoxylin and eosin and Masson Trichome staining were conducted at the Toronto Centre for Phenogenomics, (Toronto, ON). Immunostaining was conducted as described previously [21]. Antibodies used include: rabbit polyclonal anti-Syk (clone N-19; Santa Cruz Biotechnology Inc., Santa Cruz, CA) antibody, mouse monoclonal anti-α-actin (Sigma-Aldrich, Canada) and 1:400 goat polyclonal anti-Clara cell 10 protein antibody (Santa Cruz Biotechnology). DAPI was purchased from Life Technologies (Burlington, ON). Immunofluoresence images were captured using a 63 × objective on a Zeiss LSM510 Laser Scanning Confocal Microscope.

### Detection of inflammatory mediator expression

Total RNA was extracted from the lungs and purified with the RNAeasy Mini Kit. Quantitative PCR analyses were performed using the SYBR green Real Time Quantitative RT-PCR Kit (Roche Bioscience). The primer sets used are listed in Table 1.

**Table 1:** TaqMan^®^ Gene expression primers.

Gene	Assay ID
peptidylprolyl isomerase A pseudogene 8 (Ppia-ps8)	Mm00620857_s1
interleukin 6 (IL-6)	Mm00446190_m1
chemokine (C-X-C motif) ligand 1 (CXCL-1)	Mm04207460_m1
interleukin 13 (IL-13)	Mm00434204_m1
interleukin 17A (IL-17A)	Mm00439618_m1
EGF-like domain 7 (EGF-L7)	Mm00618004_m1
chemokine (C-C motif) ligand 11 (Eotaxin, CcL-11)	Mm00441238_m1
fibroblast growth factor 2 (FGF-2)	Mm00433287_m1
matrix metallopeptidase 9 (MMP-9)	Mm00442991_m1
taste receptor, type 2, member 107 (TAS2R-107)	Mm01701709_s1
taste receptor, type 2, member 108 (TAS2R-108)	Mm00498514_s1
transforming growth factor, beta 1 (TGF-beta 1)	Mm01178820_m1
tumor necrosis factor (TNF-alpha)	Mm00443258_m1

### Quantification of total collagen content

The total collagen content in the lung was assessed using the Quickzyme Total Collagen Assay Kit and normalized to total protein content using the Quickzyme Total Protein Assay Kit (both from Quickzyme Biosciences Inc, Leiden, Netherlands). The assays were conducted according to the manufacturer’s instructions. In brief, lung tissue was hydrolyzed with 6 M HCl at 95°C for 20 h and diluted 30-fold. Thirty-five μl aliquots were incubated on a microtiter plate and with 75 μl assay buffer for 20 min at room temperature. Seventy-five μl of detection reagent A and B (in a 2:3 ratio) was then added, and the plate was incubated at 60°C for 60 min. Absorbance at 570 nm was detected. Serial dilutions were conducted to ensure that samples were within the range of the standard curve.

### Quantification of HDM-specific IgE in serum

Serum HDM-specific IgE levels were detected using the antigen-capture ELISA method. Briefly, NUNC MaxiSorp^®^ plates (Sigma Aldrich Co., Mississauga, ON) were coated using 5 μg HDM in 100μl of coating buffer and incubated at 4°C overnight. Non-specific binding was blocked using 200μl/well of assay diluent. After washing, 100 μl aliquots of serum (3 serial dilutions/sample) were added and incubated at 4°C overnight. Plates were washed. 100 μl of biotin-anti-mouse IgE (BioLegend, CA, USA) was added to each well and incubated for 1 h followed by 30 min with avidin-horse radish peroxidase (BioLegend). The wells were incubated in the dark for 30 min in 100μl of TMB substrate solution (BioLegend). Optical densities were read at 450 nm with reference at 540 nm using the Titertek Multiskan^®^ Ascent spectrophotometer (Titertek Instruments Inc., Huntsville, AL, USA). To account for inter-strain variability, the data was normalized to the respective Saline Controls for each genotype.

### Preparation of *ex vivo* precision lung slices and pharmacologic inhibition

Precision-cut lung slices were prepared, as previously described [22] from BALB/c mice following the HDM sensitization or GSK treatment protocol. After 24-72h in culture, viable slices were used for experimentation. Baseline images were recorded before applying 20 mg/ml MCh to the airway lumen. Images were acquired at 100 frames/sec for 60 seconds using the NIS-Elements B3.0 imaging software (Laboratory Imaging, Czech Republic), Nikon ECLIPSE Ti-S Inverted Microscope (20x objective) and a DIGITAL-SIGHT DS-Ri1 camera (Nikon, Canada), and were stored in .tif format. Airway luminal area was measured using ImageJ (NIH, Bethesda MD) and expressed as a percent of the baseline area.

For the pharmacological inhibitor studies, lung slices were incubated with the following: PI3 kinase inhibitor (Ly294002, 50 μM, Cedarlane, ON), MLCK inhibitor (ML7, 30 μM, Sigma, Canada), Rho Kinase inhibitor (Y2763, 1 μM, Cedarlane, ON) or DMSO diluent for 30 min prior to instillation of MCh. At least 2 lung slices from each mouse were evaluated. Inhibitor and DMSO treatments were performed from serial slices obtained from the same mouse.

### Fluorescence-activated Cell Sorting (FACS) analysis

Lungs were perfused with PBS prior to excision and were then minced. Single cell suspensions were generated by digestion with collagenase type IV (0.5mg/ml) and DNAse I (250 U/ml, Sigma-Aldrich, Canada) for 30 min at 37°C in HBSS. Residual tissue was crushed in a cell strainer. Erythrocytes were removed using ammonium chloride lysis buffer, and lymphocytes were isolated, as described previously [29].

The following anti-mouse antibodies were purchased from eBioscience (San Diego, CA) or BioLegend (San Diego, CA): CD45-Brilliant Violet 510 clone 30-F11, CD5-FITC clone 53–7.3, CD19-Alexa Fluor 700 clone 6D5, IgD-Pacific Blue clone 11-26c.2a, IgM-APC/Cy7 clone RMM-1, CD23-PE clone B3B4, CD43-APC clone S11, and CD3-PE/Cy7 clone 145-2C11, CD11c-Brilliant Violet 650 clone N418. Dead cells were excluded from the analysis using the Zombie UV fixable viability kit (BioLegend), according to the manufacturer’s instructions. Cells were fixed after staining using 1% paraformaldehyde in PBS for 20 minutes prior to washing and data acquisition on an LSR II flow cytometer (BD Biosciences, Mississauga ON).

### Statistical analysis

All data are expressed as the mean±SEM. Binary comparisons were made using Student t-test. Comparisons between multiple groups were made using 1-way ANOVA, with post-hoc comparison using Dunn’s multiple-comparison test. Individual differences of the methacholine dose-response relationships were compared using two-way ANOVA [28]. All analyses were conducted using GraphPad Prism5.0 (GraphPad Software, La Jolla, CA).

## Results

### Syk is critical for the development of airway hyper-responsiveness to methacholine in the chronic HDM model of allergic airways inflammation

**Fig 2:**
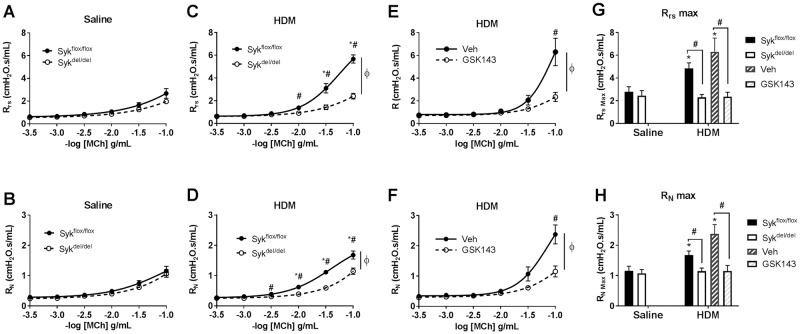
Deletion of Syk prior to HDM and inhibition of Syk activity after development of chronic allergic airways inflammation abrogates airways hyperresponsiveness to methacholine in Syk^flox/flox^//rosa26CreER^T2^ mice. In the Syk-expressing (Syk^flox/flox^) and Syk^flox/flox^ mice treated with methylcellulose control (MC: panels **A-F**), the chronic (8-week) HDM sensitization and challenge protocol increased MCh responsiveness of the total respiratory resistance (R_rs_, **A, C, E**) and central airways Newtonian resistance (R_N_, **B, D, F**) in a dose-dependent manner compared with the Saline-exposed group (*, p<0.05, n = 8-10/group). However, HDM failed to augment MCh responsiveness in the Syk-deleted state (Syk^del/del^; **C,D**; open circles) or in Syk^flox/flox^ mice treated with GSK143 (**E, F**; open circles). These responses were most obvious at the maximum R_rs_ and R_N_ (**G,H**, *p<0.05 HDM vs. Saline, same genotype; #p<0.05, Syk^flox/flox^ vs. Syk^del/del^ and Veh vs. GSK143; same exposure group, n = 8-10/group).

In the chronic 8-week HDM model of allergic airways inflammation protocol, we observed enhanced MCh responsiveness of the total respiratory system (R_rs_) and central airways Newtonian resistance (R_N_) in Syk-expressing, Syk^flox/flox^ mice compared with non-sensitized saline-exposed controls; deletion of Syk prior to HDM-sensitization and challenge prevented development of AHR, as indicated by the attenuated R_rs_ and R_N_ in the Syk^del/del^ compared with the Syk^flox/flox^ mice (Fig 2A–2D *p<0.05, HDM vs.Saline control, same genotype; #p<0.05, Syk^flox/flox^ vs. Syk^del/del^, HDM group; ɸ *p<0.05, two-way ANOVA for the dose-response curve, n = 8-10/group). To determine whether inhibition of Syk can attenuate AHR once the disease phenotype is established, we treated the non-induced, Syk-expressing Syk^flox/flox^//rosa26CreER^T2^ mice with GSK143 (30 mg/kg) or methyl cellulose vehical control (Veh) daily for 2 weeks *after* completion of the 8-week HDM exposure period. As shown in Fig 2E and 2F, treatment with GSK143 significantly reduced both R_rs_ and R_N_ in the HDM mice to levels observed in the Saline controls #p<0.05, Syk^flox/flox^ vs. Syk^del/del^, HDM group; ɸ p<0.05, two-way ANOVA for the dose-response curve, n = 8-10/group). The most obvious difference were observed for the maximal R_rs_ and R_N_ (Fig 2G and 2H; *p<0.05 compared with Syk^flox/flox^:HDM; #p<0.05, Syk^flox/flox^ vs. Syk^del/del^ and Veh vs. GSK143 n = 8-10/group).

**Fig 3:**
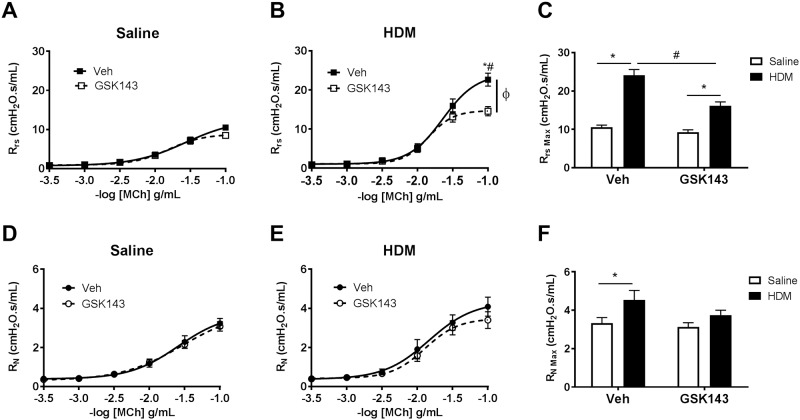
Daily treatment with GSK143 for 2 weeks *following* chronic HDM sensitization abrogates airways hyperresponsiveness to methacholine in BALB/c mice. BALB/c mice underwent an 8-week HDM sensitization and challenge protocol followed by 2 weeks of treatment with GSK143 or Veh control. (**A-C**) HDM exposure increased MCh responsiveness of the total respiratory resistance (R_rs_) compared with the Saline-exposed mice treated with MC; this was most evident at the maximal R_rs_. HDM mice subsequently treated with GSK143 exhibited increased MCh responsiveness when compared naïve Saline-exposed mice; however, this response was significantly attenuated compared with HDM mice treated with MC. ϕ, p<0.05 dose response curve; *p<0.05 HDM vs. Saline, same treatment group; #p<0.05 GSK143 vs. Veh; same exposure group, n = 11-14/group. (**D**-**F**) HDM increased R_N_ in the Veh treated group, but no differences between the VEH and GSK143 mice groups were observed. *p<0.05 HDM vs. Saline, same treatment group, n = 11-14/group.

Strain variation to allergen-induced AHR has been well described [30,31]. To further characterize the role of Syk in allergen-induced AHR, we conducted parallel studies in BALB/c mice, a strain that is known to readily develop allergic airways inflammation and AHR. Following the 8-week HDM exposures, BALB/c mice were treated with GSK143 (30 mg/kg) or Veh daily for 2 weeks using the same protocol as that for the Syk^flox/flox^//rosa26CreER^T2^ mice. HDM sensitization and challenge resulted in enhanced MCh responsiveness of the R_rs_ compared with Saline-exposed mice in the MC-treated control group; this was most evident at the maximal R_rs_ (Fig 3A–3C, *p<0.05, n = 11-14/group). HDM mice that were subsequently treated with GSK143 (i.e., in the treatment of established disease) exhibited attenuated R_rs_ compared with the Veh controls; differences were observed for both the dose-response curve and maximal R_rs_ (Fig 3B and 3C, ɸ p<0.05, dose-response curve; *p<0.05, HDM vs. Saline; #p<0.05, GSK143 vs. MC, n = 11-14/group). HDM also increased R_N_ in the Veh group, but no difference was observed between the GSK143- and Veh-treated mice (Fig 3D–3F, *p<0.05, HDM vs. Saline, n = 11-14/group).

### Loss of Syk activity does not suppress circulating leukocytes and preferentially attenuates neutrophil recruitment to the airways following HDM sensitization and challenge

**Fig 4:**
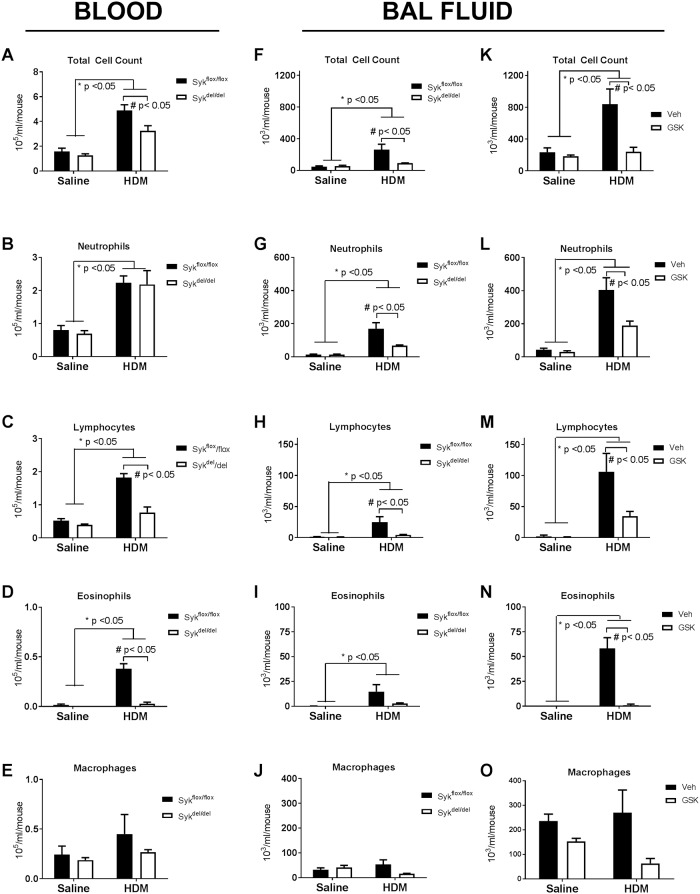
Effect of loss of Syk activity on peripheral blood and bronchial alveolar lavage fluid total and differential leukocyte counts under basal conditions and following 8 weeks of HDM sensitization and challenge. **A-E:** At 8 weeks after treatment with tamoxifen, as described in Fig 1, no significant differences in the total or differential blood leukocyte counts were observed between Syk^flox/flox^ and Syk^del/del^ mice in the Saline groups. Furthermore, 8 weeks of HDM sensitization and challenge resulted in significant peripheral leukocytosis in both groups of mice, with Syk deletion impairing increases in the peripheral lymphocyte and eosinophil counts (*p<0.05, Saline vs. HDM; #p<0.05, Syk^flox/flox^ vs. Syk^del/del^, n = 4/group). **F-J:** HDM significantly increased BAL fluid total cell, neutrophil, lymphocyte and eosinophil cell counts compared with Saline mice (*p<0.05, 2-way ANOVA, n = 5-6/group); within the HDM mice, deletion of Syk significantly impaired recruitment of neutrophils and lymphocytes (#p<0.05, Syk^flox/flox^ vs. Syk^del/del^, n = 5-6/group). **K-O:** BALB/c mice exhibited a more pronounced BAL fluid leukocytosis following HDM exposure when compared with Syk^flox/flox^//rosa26CreER^T2^ mice; however, the pattern of response was similar. HDM lead to increases in the total BAL fluid cell counts, with significant increases in neutrophils, lymphocytes and eosinophils in the Veh control group, a reponse that was attenuated by treatment with GSK143 (*p<0.05, Saline vs. HDM, 2-way ANOVA; # p<0.05, Veh vs. GSK143, n = 5-6/group).

Syk plays a critical role in hematopoietic cell proliferation and maturation. Thus, we evaluated the effect of Syk deletion on peripheral and BAL leukocyte counts. Under normal basal conditions (Saline control), deletion of Syk exhibited no effect on the total or differential peripheral leukocyte counts (Fig 4A–4E). Despite Syk deficiency at the initiation of HDM sensitization, the 8-week exposure elicited peripheral leukocytosis in both the Syk^del/del^ and Syk^flox/flox^ mice, and was accompanied by significant increases in the total numbers of neutrophils, lymphocytes and eosinophils (Fig 4A–4D; * p <0.05, Saline vs. HDM, n = 4/group). Among the HDM-exposed groups of mice, the Syk^del/del^ mice exhibited significantly lower total leukocyte counts, lymphocytes and eosinophils compared with the Syk^flox/flox^ mice (Fig 4A, 4C and 4D; # p <0.05, Syk^del/del^ mice vs. Syk^flox/flox^ mice, n = 4/group). However, no differences were observed in the peripheral neutrophil counts between the Syk^flox/flox^ and Syk^del/del^ mice following HDM sensitization and challenge (Fig 4B).

In the BAL fluid, HDM sensitization and challenge led to significant increases in total leukocytes, neutrophils, lymphocytes and eosinophils in both Syk^flox/flox^ and Syk^del/del^ mice (Fig 4F–4J, *p< 0.05, HDM vs. Saline, n = 5-6/group). However, among the HDM-exposed mice, loss of Syk resulted in significantly lower total BAL fluid leukocytes, neutrophils and lymphocytes (Fig 4F–4H; #p<0.05, Syk^del/del^ vs. Syk^flox/flox^, HDM group; n = 5-6/group). There was a tendency toward decreased BAL fluid eosinophils in the HDM-exposed Syk^del/del^ mice compared with the HDM-exposed Syk^flox/flox^ but this difference was not statistically significant. (Fig 4I).

A similar pattern of leukocyte recruitment to the BAL fluid was observed in the HDM-sensitized BALB/c mice; although the magnitude of inflammatory cell recruitment, like that of the MCh-responsiveness, was higher than the Syk^flox/flox^//rosa26CreER^T2^ mice. Compared with the Saline controls, HDM-exposed mice exhibited increases in the BAL fluid total leukocyte counts, with significant increases in neutrophils, lymphocytes and eosinophils; this was observed in both MC- and GSK143-treated mice (Fig 4K–4O, *p<0.05, HDM vs. Saline, n = 5-6/group). GSK143 had no significant effect on the total or differential cells counts in the BAL of the Saline-exposed mice. However, among the HDM-exposed mice, GSK143 treatment attenuated the HDM-induced increases in the BAL fluid total cell counts, neutrophils, lymphocytes and eosinophils (Fig 4K–4N, #p<0.05, Veh vs. GSK, HDM group, n = 5-6/group).

Although loss of Syk had no effect on peripheral neutrophil counts in the Saline and HDM groups, neutrophil recruitment to BAL fluid was significantly impaired in HDM-exposed mice, suggesting that loss of Syk activity preferentially impaired neutrophil recruitment to the airways in response to HDM sensitization, as decreases of lymphocytes and eosinophils in the BAL fluid were likely due to the low numbers of these leukocytes sub-populations in the peripheral blood.

FACS analysis of leukocytes isolated from digested lungs did not reveal significant differences in the proportions of major T, B, or CD11c+ cell populations between HDM and saline exposure or between Veh and GSK treatment (S1A–S1C Fig).

### Loss of Syk activity does not affect airway inflammation and remodeling in the chronic HDM model

**Fig 5:**
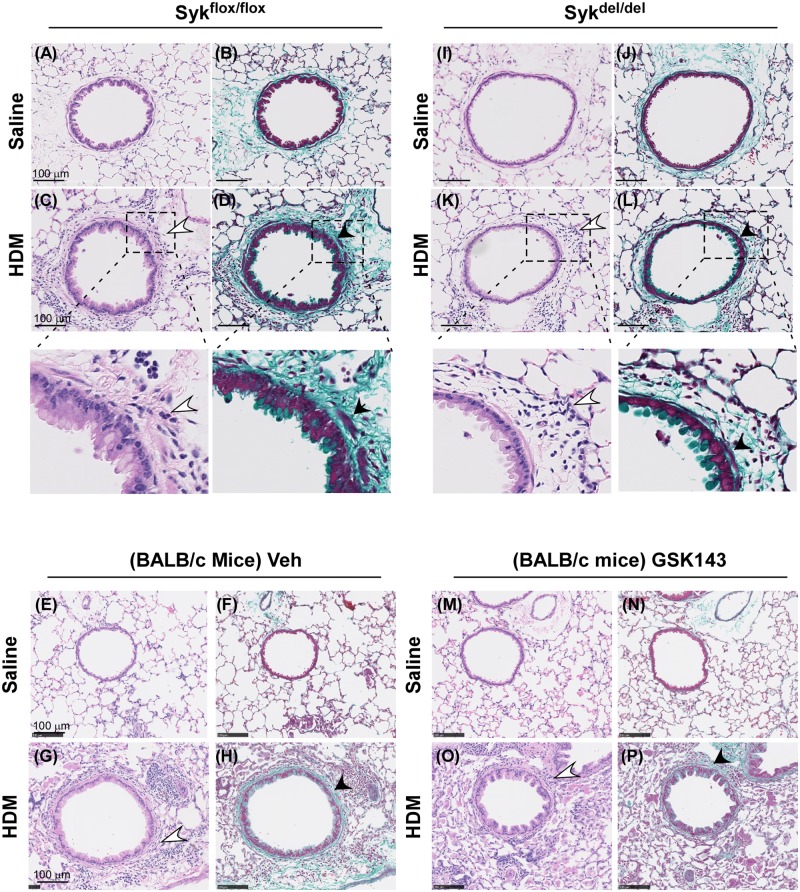
Loss of Syk activity does not abolish airway recruitment of inflammatory cell in the chronic HDM-challenge model. A-H: Hematoxylin and eosin staining of lung sections revealed inflammatory cell peribronchial infiltrates (open arrows; **C and inset, G**) while Trichome Masson staining showed increased epithelial and basement membrane thickening and smooth muscle cell hypertrophy (closed arrows, **D and inset, H**; closed arrows) in the airways of HDM compared with Saline-exposed mice in the Syk^flox/flox^ group and BALB/c mice treated with Veh control. Within the HDM groups of mice, the Syk^del/del^ (**K,L**) and BALB/c mice treated with GSK143 (**O,P**) did not exhibit significant differences in the inflammatory infiltrate, basement membrane thickening nor smooth muscle cell hypertrophy when compared with their respective Syk^flox/flox^ (**C,D**) and MC-treated controls. (**G.H**). No differences were noted amongst the 4 Saline-exposed groups (**A,B, I,J, E,F, M,N**). Images are representative of 4 mice/group. Bar represents 100 μm.

Histological examination (Fig 5) revealed significant inflammatory peribronchial cellular infiltrates (open arrow) and increased collagen accumulation (solid arrow) in the airways of HDM (C,D,K,L and insets; G,H) compared with Saline-exposed mice (A,B,I,J,E,F,M,N). In the HDM mice, the accumulation of inflammatory cell infiltrates and collagen deposition did not appear to be significantly different in either the Syk^del/del^ mice, when Syk deletion occurred prior to sensitization (K,L), nor in the GSK-treated mice when the Syk inhibitor was given after sensitization (O,P) compared with their Syk^flox/flox^ (C,D) and Veh controls (G,H), respectively. No effect of either Syk deletion or inhibition was observed in the Saline mice (Fig 5A, 5B, 5I, 5J, 5E, 5F, 5M and 5N).

**Fig 6:**
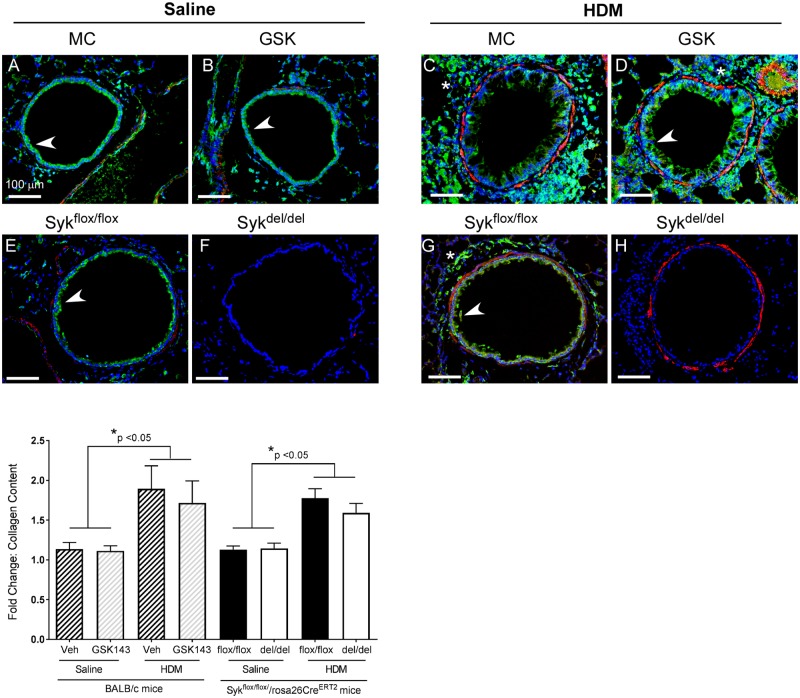
Loss of Syk activity does not attenuate airway inflammation and remodeling or total lung collagen content in the chronic HDM-challenge model. Immunostaining for Syk (green) revealed expression in airway epithelia (arrow) and leukocytes (*) under basal Saline conditions and following HDM exposure in all groups of mice (**A-E, G**) except the Syk-deficient (Syk^del/del^) mice (**F, H**). Following HDM exposure, increased peribronchial leukocyte recruitment (DAPI staining, blue) was also observed (**C,D,G,H**), as was an increase in α-smooth muscle actin (stained red); this pattern was observed in the control Veh and Syk^flox/flox^ mice (**C,G**) as well as the GSK-treated and Syk-deficient Syk^del/del^ mice (**D,H**). Images are representative of 4 mice/group. Bar represents 100 μm. **I:** Quantitative analysis revealed a 2-fold increase in the total lung collagen content in the HDM mice when compared with Saline control in all groups of mice (*p<0.05, HDM vs. Saline, 2 way ANOVA, n = 4/group). No differences were observed between Syk^flox/flox^ and Syk^del/del^ mice, nor between BALB/c mice treated with Veh or GSK143.

Subsequent immunostaining (Fig 6) showed significant increases in α-smooth muscle staining (red) in the peribronchial regions of the HDM- (C,D,G,H) compared with the Saline-exposed mice (A,B,E,F); this was evident in both the GSK143 and MC-treated (C,D) as well as the Syk^flox/flox^ and Syk^del/del^ (G,H) mice. Immunostaining for Syk showed expression in airway epithelia (arrow) and inflammatory cells (*) in Syk^flox/flox^ mice and BALB/c mice, regardless of Veh or GSK143 treatment (A-D, E,G). As expected, no Syk expression was detected in the Syk^del/del^ mice (F,H) despite histological evidence of peribronchial inflammatory cell infiltration (indicated by the DAPI stain, blue). Thus, the histological analyses corroborate our findings in the BAL fluid cell counts: Syk activity is not essential for overall leukocyte recruitment following HDM sensitization and challenge.

To better quantify collagen content in the lung, we measured the total collagen content using a spectrophotometric assay. As shown in Fig 6I, in both BALB/c and Syk^flox/flox^//rosa26CreER^T2^ mice, 8 weeks of exposure to HDM led to a two-fold increase in collagen content in the lungs compared with Saline control mice (p<0.05, n = 4/group) regardless of the presence or absence of Syk expression or activity. Among the HDM groups, no differences in the collagen content were observed between the GSK143-treated and the MC-treated control mice (p>0.05, n = 4); similarly, there were no differences between the HDM- exposed Syk^flox/flox^ and Syk^del/del^ mice (p>0.05).

### Loss of Syk activity does not abolish the allergic response to HDM

**Fig 7:**
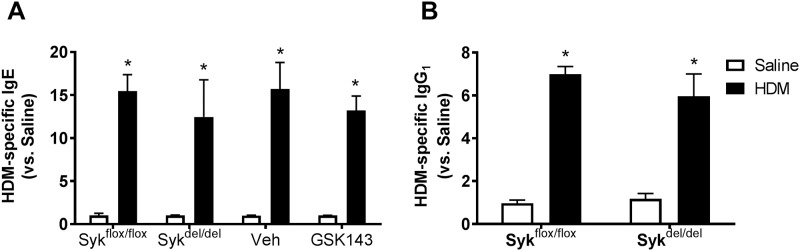
Loss of Syk activity attenuates but does not abolish production of HDM-specific IgE and IgG_1_ in response to HDM-sensization and challenge. HMD sensitization and challenge resulted in significant increases in the serum concencrations of HDM-specific IgE (**A**) and IgG_1_ (**B**) compared with Saline control (*p< 0.05, n = 6-9/group). In the HDM-exposed mice, no differences were observed between the Syk^del/del^ and Syk^flox/flox^ mice nor between the BALB/c mice treated with GSK143 and Veh controls.

Syk plays a critical role in Fcε receptor signaling and the allergic response. To determine whether deletion of Syk *prior to* or treatment with Syk inhibitor *after* HDM sensitization affected the allergic response, we measured HDM-specific serum IgE levels. Both Syk^flox/flox^ and Syk^del/del^ mice were able to mount a significant IgE response to HDM compared with Saline-control mice; similarly, treatment with GSK143 for 2 weeks following completion of HDM sensitization did not affect the HDM-specific IgE response (Fig 7A, *p<0.05 HDM vs. Saline, n = 5-6/group). Similar responses were observed for HDM-specific IgG_1_ in sensitized Syk^flox/flox^ and Syk^del/del^ mice (Fig 7B, *p<0.05 HDM vs. Saline, n = 5-6/group). Thus, HDM mice showed an appropriate allergic response after sensitization to HDM.

### Loss of Syk activity exhibits differential effects on inflammatory mediator expression

**Fig 8:**
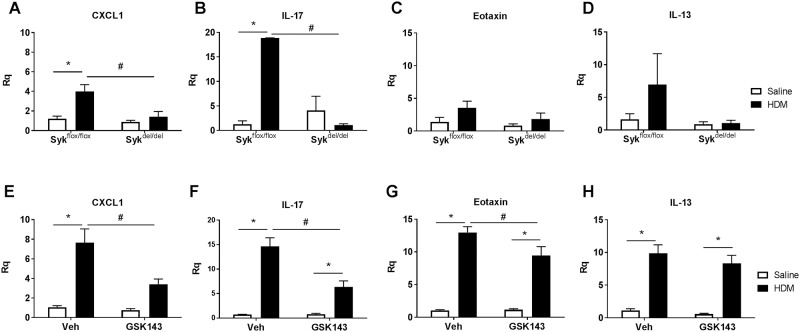
Syk regulates expression of CXCL-1 and IL-17 expression in the chronic HDM- model of allergic airways inflammation. HDM sensitization and challenge for 8 weeks increased expression of CXCL-1 and IL-17, a response that was significantly abrogated in Syk^del/del^ mice (**A,B**) and following treatment with GSK143 (**E,F**). HDM-exposure induced the expression of eotaxin (**C,G**). Furthermore, a role for Syk in modulating this response was observed in the BALB/c mice treated with GSK143 (**G**), although no differences were observed between the Syk^flox/flox^ and Syk^del/del^ mice (**C**). Increased IL-13 expression following HDM exposure was observed (**D,H**); however, a role of Syk in regulating this response was not consistently observed in the BALB/c and Syk^flox/flox^//rosa26CreER^T2^ mice. *p< 0.05, HDM vs. Saline, n = 5-6/group; # p<0.05, Syk^flox/flox^ vs. Syk^del/del^, n = 5-6/group.

To better understand the role of Syk in mediating the pulmonary immune response to HDM, we analyzed inflammatory mediator gene expression using qPCR, focusing on those implicated in the pathogenesis of allergic asthma and/or previously reported to be regulated by Syk [21,32–34]. HDM sensitization significantly increased several chemokines and cytokines in Syk^flox/flox^ and MC-treated BALB/c mice, including the neutrophil chemokine, CXCL-1, and the pro-inflammatory cytokine, IL-17, as well as eotaxin and IL13 (Fig 8, *p<0.05 HDM vs. Saline, n = 5-6/group). Of these, CXCL-1 and IL-17 were found to be Syk-dependent, as their expression in Syk^del/del^ and GSK143-treated mice was significantly impaired compared with their respective controls (Fig 8A, 8B, 8E and 8F, #p<0.05, HDM group, n = 5-6/group). Eotaxin expression was significantly attenuated in GSK143-treated lungs, but was not significantly different between the Syk^flox/flox^ and Syk^del/del^ groups (Fig 8C and 8G #p<0.05, HDM group, n = 5-6/group). The HDM-induced increase in IL13 expression showed a tendency toward Syk-dependence in the Syk^del/del^ mice, but this was not statistically significant compared with the Syk^flox/flox^, and was not apparent in the GSK- and MC-treated groups (Fig 8D and 8H, p = 0.08, n = 5-6/group). Other mediators, including IL-6, RANTES, VEGF and TNF-α, were increased following HDM sensitization but no significant differences were observed between Syk^flox/flox^ and Syk^del/del^ groups nor in the GSK143- and MC-treated mice (data not shown). Measurement of KC and IL-17 protein levels in BAL from Syk^flox/flox^ and Syk^del/del^ mice were consistent with the gene expression findings (data not shown).

### Syk regulates airway contraction *ex vivo* in the chronic model of allergic airways inflammation

**Fig 9:**
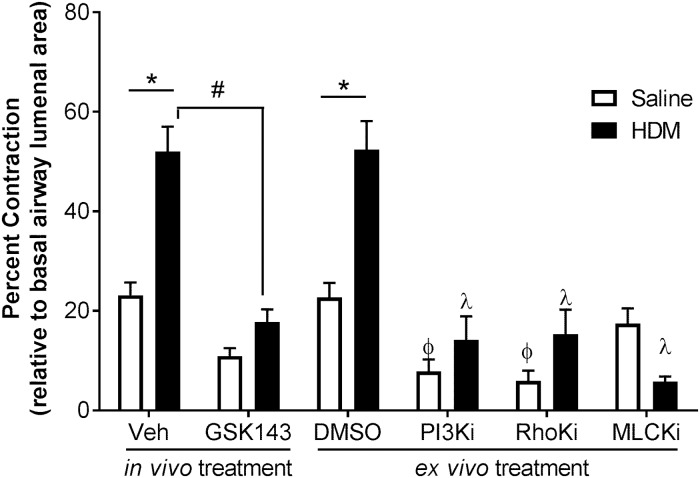
Treatment with GSK143 impairs airway contractility to a similar degree as known inhibitors of smooth muscle contraction. Precision cut lung slices were prepared from BALB/c mice following HDM (solid bars) or Saline exposure (open bars), and the MCh-induced airway contraction was measured. Airways from HDM-exposed mice showed significantly increased airway contraction compared with Saline-mice in the Veh-treated group (*p< 0.05, n = 12 /group), which was abolished by treatment with GSK143 (# p<0.05, n = 12/group). In the HDM mice (solid bars), incubation of the lung slices for 30 mins with inhibitors of PI3 kinase (50 μM), RhoK (1 μM) and MLCK (30 μM) significantly impaired airway contraction compared with slices incubated with DMSO, the diluent control (λ, p<0.05, n = 9-19/group); impairment of airway contraction was similar to that observed in the GSK143-treated airways. Within the DMSO group, HDM-exposed airways showed significantly increased airway contractility compared with Saline-exposed airways (*p<0.05, n = 9-19/group). In the airways of Saline-mice (open bars), inhibitors of PI3 kinase and RhoK significantly impaired airway contraction compared with DMSO-treated airways (ϕ, p<0.05, Saline group, n = 9-19/group), suggesting that these mediators play a role in maintaining normal airway tone.

We previously reported that Syk regulates airway contractility in normal healthy mice, and that this occurs independent of its role in mediating immune cell activity [22]. To determine whether Syk plays a similar role in the chronically, allergically inflamed airways, precision-cut lung slices were prepared from BALB/c mice after the 8-week HDM exposure period (Fig 9). As expected, airway slices from HDM-sensitized mice (solid bars) exhibited enhanced contraction *ex vivo* in response to MCh compared with airways from Saline-exposed mice (open bars) in both the Veh and DMSO control groups (*p<0.05, n = 12 slices/group). *Ex vivo* contractility to MCh was significantly reduced in airways of HDM mice treated with GSK143, compared with those treated with Veh (#p<0.05, n = 12 slices/group); this was similar to those observed in MC-treated Saline mice.

Lastly, to further understand the mechanisms underlying the role of Syk in airways contractility, we treated the airway slices *ex vivo* with inhibitors to known mediators of smooth muscle contraction, including myosin light chain kinase (MLCKi), Rho kinase (RhoKi) and phosphatidylinositol-3 kinase (PI3Ki). Treatment with all three inhibitors resulted in significant attenuation of airway contractility when compared with DMSO control (Fig 9, λ p<0.05, n = 9–19 slices/group) in the HDM-sensitized mice. In the Saline group, PI3K and RhoK inhibitors further reduced MCh-induced airway contraction compared with DMSO control, suggesting that these kinases are important for maintaining basal airway tone (Fig 9, ϕ p<0.05, n = 9–19 slices/group).

## Discussion

In this report, we employed Syk^flox/flox^//rosa26CreER^T2^ Syk knock-out mice to investigate the role of Syk in the *development* of AHR and airway inflammation in an 8-week model of HDM-induced allergic airways inflammation. BALB/c mice treated with GSK143 for 2 weeks *after* an 8-week HDM-sensitization and challenge protocol were used to evaluate the effect of Syk inhibition in *reversing* AHR and airway inflammation after establishment of allergic airways inflammation (i.e., in a treatment-based protocol). In both Syk^flox/flox^ and wild-type BALB/c mice, 8 weeks of HDM exposure resulted in AHR, with enhanced R_rs_ and R_N_ to MCh. AHR was accompanied by BAL neutrophilia and up-regulated expression of CXCL-1, a neutrophil chemokine, and IL-17, a pro-inflammatory cytokine that is known to recruit neutrophils indirectly via IL-8, to sites of inflammation. Different strains of mice are known to respond to allergen sensitization and challenge with varying degrees of airways inflammation and AHR [30,31]. While the magnitudes of the increases in R_rs_ and R_N_ following 8 weeks of HDM exposure were different between the BALB/c and Syk^flox/flox^//rosa26CreER^T2^ strains of mice, the increases exhibited Syk-dependence in both strains. Loss of Syk activity prior to, and treatment with Syk inhibitors following HDM exposure attenuated AHR, BAL neutrophilia and expression of both CXCL-1 and IL-17. A tendency toward BAL lymphocytosis and eosinophilia in response to HDM was not consistently observed; similarly, a trend to decreased BAL lymphocyte and eosinophil cell counts following deletion of Syk or treatment with GSK143 was not statistically significant.

Syk is necessary for B lymphocyte maturation and proliferation [35]. Our previous studies in a mouse trachea allograft transplant model demonstrated that Syk plays a role in the allo-immune response by preferential recruitment of B lymphocytes [29]. To determine whether differential T and B lymphocyte recruitment occurred in HDM-induced allergic airways inflammation, we conducted FACS analysis of isolated pulmonary leukocytes. We observed no significant differences in the proportions of CD3+ (a T cell marker) or CD19+ (a B cell marker) cells amongst the different experimental groups (S1A Fig). Although Syk has been implicated in dendritic cell activity in the acute model of allergic airways inflammation [20], we did not find significant differences in the proportions of CD11c+ (a monocyte/macrophage and dendritic cell marker) amongst the different groups (S1A Fig). Subset analysis of specific B-lymphocyte populations also showed no significant differences between the MC- and GSK143-treated groups, or between the HDM and Saline groups (S1B and S1C Fig). Thus, our observations indicate that Syk mediates neutrophilic airway inflammation and AHR in the chronic model of HDM-induced allergic airways inflammation.

Studies using clinical cluster analysis with large cohorts have recently identified four to five clinical sub-phenotypes of asthma, based on age of disease onset, severity of asthma, response to therapy and presence of eosinophilia and/or neutrophilia [10,13,14,36,37]. Neutrophilic asthma is associated with increased disease severity, systemic inflammation and poor response to therapy [10,38–41]. While representing a small fraction of asthmatic patients, drug-resistant asthma is associated with disproportionate morbidity and disease burden; no effective therapy has yet been developed to improve the outcome in this subpopulation. Our observations in the chronic HDM model suggest that neutrophilic AHR and airway inflammation can be attenuated by inhibition of Syk, even after establishment of the asthmatic phenotype. However, airway remodeling with evidence of collagen deposition and increased α-smooth muscle actin deposition, occurs despite the absence of Syk activity, attenuation of neutrophilia and expression of CXCL-1 and IL-17. Interestingly, our *ex vivo* lung slice studies revealed that inhibition of Syk effectively reduced MCh-induced airway contraction to the same extent as inhibition of the signaling pathways that regulate smooth muscle contraction (Fig 9). This response was observed after up to 96 h of *ex vivo* culture in the absence of inflammatory cells, suggesting that this Syk-mediated response occurs independent of leukocyte function.

We previously reported preferential upregulation of Syk expression in airway epithelia in a chronic ovalbulmin-induced model of allergic airways inflammation (24). Our current study adds to these observations by demonstrating additional roles for Syk in modulation of the neutrophilic asthma phenotype by mediating airways contractility and the augmented responsiveness to methacholine in the chronic HDM model of allergic airways inflammation. Thus, Syk inhibitors could play a role in the asthma armamentarium as a potential adjunct therapy in combination with other drugs in the management of neutrophilic asthma.

## Supporting Information

S1 Fig **(A)**. FACS analysis of isolated pulmonary leukocytes demonstrated no significant differences in the proportions of CD3+, CD19+ cells or CD11c+ cells amongst the different experimental groups. (**B,C**) Subset analysis of specific B-lymphocyte populations also showed no significant differences between the MC- and GSK143-treated groups, or between the HDM and Saline groups (n = 3/group).
(TIF)
