# Supplementary figures and images for: Syk Regulates Neutrophilic Airway Hyper-Responsiveness in a Chronic Mouse Model of Allergic Airways Inflammation
Source: PLoS One. 2017 Jan 20;12(1):e0163614. doi: 10.1371/journal.pone.0163614 (PMC5249072; doi:10.1371/journal.pone.0163614)

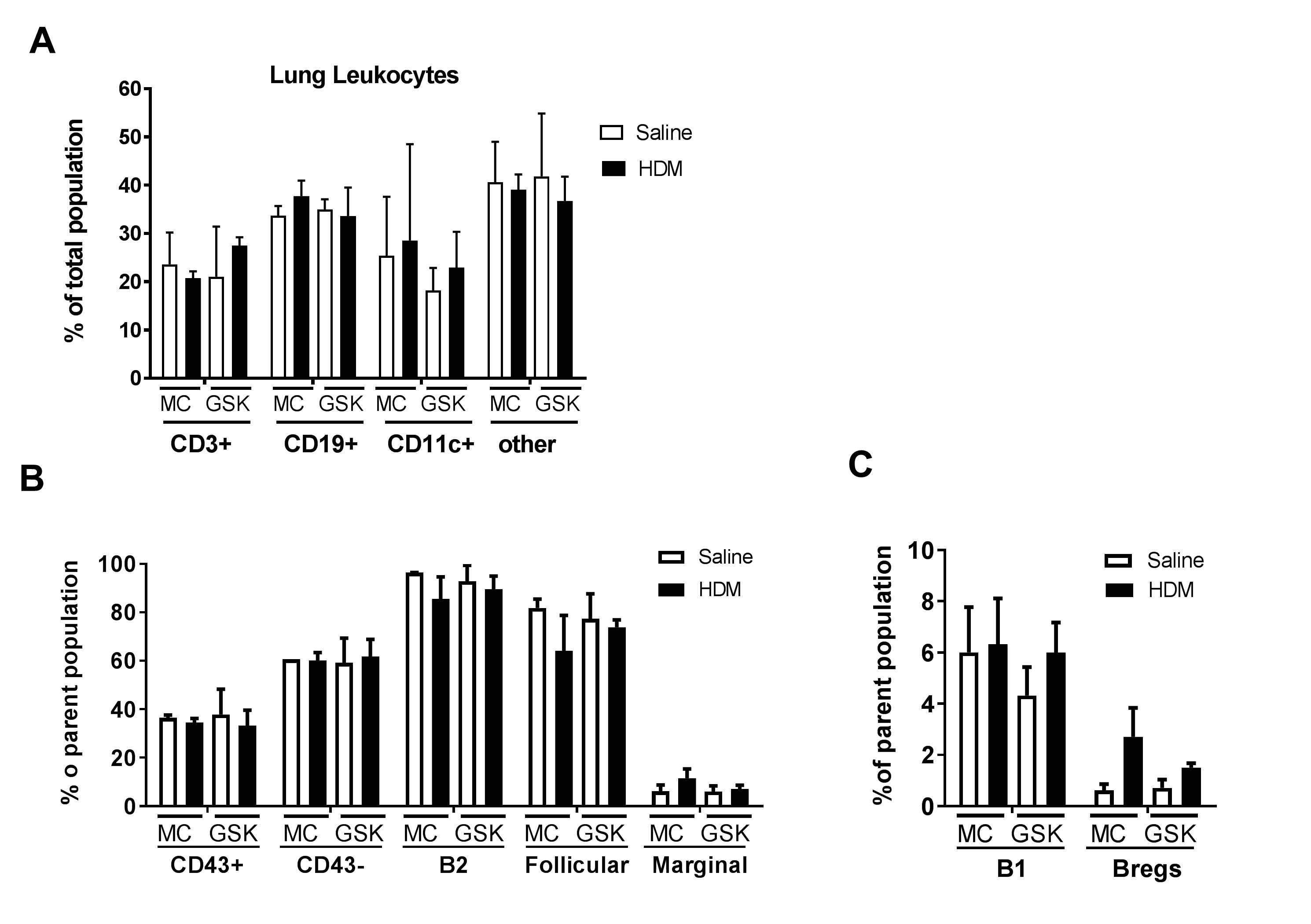

Supplement: S1 Fig — (A). FACS analysis of isolated pulmonary leukocytes demonstrated no significant differences in the proportions of CD3+, CD19+ cells or CD11c+ cells amongst the different experimental groups. (B,C) Subset analysis of specific B-lymphocyte populations also showed no significant differences between the MC- and GSK143-treated groups, or between the HDM and Saline groups (n = 3/group). (TIF) [file pone.0163614.s001.tif]
